# Effect of Cognitive Behavioral Therapy for Insomnia on Insomnia Symptoms for Individuals With Type 2 Diabetes: Protocol for a Pilot Randomized Controlled Trial

**DOI:** 10.2196/14647

**Published:** 2019-12-19

**Authors:** Mohammed M Alshehri, Aqeel M Alenazi, Jeffrey C Hoover, Shaima A Alothman, Milind A Phadnis, Jason L Rucker, Christie A Befort, John M Miles, Patricia M Kluding, Catherine F Siengsukon

**Affiliations:** 1 University of Kansas Medical Center Lenexa, KS United States; 2 Jazan University Jazan Saudi Arabia; 3 University of Kansas Medical Center Kansas City, KS United States; 4 Prince Sattam Bin Abdulaziz University Alkharj Saudi Arabia

**Keywords:** insomnia, type 2 diabetes, cognitive behavioral therapy, sleep variability, self-care, fatigue

## Abstract

**Background:**

Insomnia symptoms are a common form of sleep difficulty among people with type 2 diabetes (T2D) affecting sleep quality and health outcomes. Several interventional approaches have been used to improve sleep outcomes in people with T2D. Nonpharmacological approaches, such as cognitive behavioral therapy for insomnia (CBT-I), show promising results regarding safety and sustainability of improvements, although CBT-I has not been examined in people with T2D. Promoting sleep for people with insomnia and T2D could improve insomnia severity and diabetes outcomes.

**Objective:**

The objective of this study is to establish a protocol for a pilot randomized controlled trial (RCT) to examine the effect of 6 sessions of CBT-I on insomnia severity (primary outcome), sleep variability, and other health-related outcomes in individuals with T2D and insomnia symptoms.

**Methods:**

This RCT will use random mixed block size randomization with stratification to assign 28 participants with T2D and insomnia symptoms to either a CBT-I group or a health education group. Outcomes including insomnia severity; sleep variability; diabetes self-care behavior (DSCB); glycemic control (A_1c_); glucose level; sleep quality; daytime sleepiness; and symptoms of depression, anxiety, and pain will be gathered before and after the 6-week intervention. Chi-square and independent *t* tests will be used to test for between-group differences at baseline. Independent *t* tests will be used to examine the effect of the CBT-I intervention on change score means for insomnia severity, sleep variability, DSCB, A_1c_, fatigue, sleep quality, daytime sleepiness, and severity of depression, anxiety, and pain. For all analyses, alpha level will be set at .05.

**Results:**

This study recruitment began in February 2019 and was completed in September 2019.

**Conclusions:**

The intervention, including 6 sessions of CBT-I, will provide insight about its effect in improving insomnia symptoms, sleep variability, fatigue, and diabetes-related health outcomes in people with T2D and those with insomnia symptoms when compared with control.

**Trial Registration:**

ClinicalTrials.gov NCT03713996; https://clinicaltrials.gov/ct2/show/NCT03713996

**International Registered Report Identifier (IRRID):**

DERR1-10.2196/14647

## Introduction

### Background

Type 2 diabetes (T2D) is the predominant form of diabetes mellitus that results in multiple complications, including sleep difficulties [1]. It is a global health issue primarily affecting older adults [2]. It results from relative insulin deficiency and peripheral insulin resistance [3]. Consequently, T2D causes abnormal amounts of glucose in the bloodstream [4]. As a result, T2D has been linked to several complications including hyperglycemia, which may also affect multiple organs and systems [5]. As a result, hyperglycemia may lead to sleep disturbances because of associated symptoms, including headache, increased thirst, and nocturia [6].

Sleep disturbances have been shown to increase activation of the hypothalamic-pituitary-adrenal (HPA) axis [7], which may further exacerbate the management of T2D [8]. During a night of poor sleep, cortisol levels increase because of hyperactivation of the stress system *HPA*, which then leads to an increased glycation level in the blood stream [9]. As individuals with T2D are particularly susceptible to hyperglycemia, an increased glycation level may be particularly problematic [10]. To illustrate that, increasing the glucose level during a night of sleep in people with T2D may increase the bathroom visits and the number of awakenings [11]. Increasing the number of awakenings during a night of sleep is a part of poor sleep quality [12], which may further contribute in activation of the stress system [13]. This might suggest a bidirectional relationship between sleep disturbances and hyperglycemia [14]. Compounding this issue even further, previous research has shown that rates of several sleep disorders including obstructive sleep apnea, insomnia, and restless leg syndrome (RLS) are increased in people with T2D [15-17]. After controlling for age and gender, the prevalence of insomnia diagnosis is significantly higher in people with T2D, compared with those without it [15,17,18].

Insomnia is one of the most common sleep disorders in people with T2D, as more than half of their population report insomnia symptoms [17,18]. In a study of people with T2D, 8% to 17% reported difficulty falling asleep, 23% to 40% reported difficulty staying asleep, and 26% to 43% reported difficulty in both initiating and maintaining sleep [17]. In another study of 7239 individuals with T2D, 76.8% of that sample reported experiencing insomnia symptoms regularly. For those 7239 individuals, the 3 most prevalent insomnia symptoms were nocturia (43.8%), difficulty falling asleep (30.5%), and waking after sleep onset (WASO; 27.0%) [15,17].

For adults and older adults diagnosed with clinical insomnia, there are several negative effects of insomnia that are harmful to long term health, such as increases in daytime sleepiness, fall risk, fatigue, and a decline in the quality of life [19,20]. Furthermore, studies have reported that insomnia is associated with hypertension, diabetes, and cardiovascular disease [20-22]. Consequently, insomnia increases the risk of all-cause mortality 3-fold over a 15-year follow-up period [23].

Although individuals with T2D or insomnia are at increased risk of negative health outcomes, there are also unique risks to those who have both T2D and insomnia. People with T2D who experience poor sleep quality or excessive daytime sleepiness show decreased adherence to diabetes self-care behavior (DSCB) [24]. DCSB is essential in maintaining or attaining glycemic control (A_1c_) in people with T2D [25]. Sleep quality and low sleep variability are also important for well-being and a healthy life [26,27]. Indeed, poor health and quality of life are thought to be associated with poor sleep quality in people with T2D [28-30]. In addition to deficits in sleep quality, high sleep variability is common in people with insomnia [31] and, may be, even more prominent in people with T2D [32]. Furthermore, it has been found that variability of bedtime and wake time is associated with a high level of the inflammatory biomarker called tumor necrosis factor (TNF)-alpha in people with and without insomnia [33]. TNF-alpha is associated with vascular diseases, such as atherosclerosis [34].

T2D and insomnia have a bidirectional relationship, which might be because of shared risk factors [8]. Risk factors that are commonly reported by people with both T2D and insomnia include depression, anxiety, pain, and obesity [8,19,35,36]. These health issues may exacerbate the severity of insomnia symptoms, and they may add complexity to A_1c_ [37,38]. Although several studies have examined the complex relationship between T2D and insomnia while controlling for risk factors, the underlying mechanisms of this relationship are still under investigation. Although this investigation is still in its infancy, examining the effect of treating insomnia symptoms may reveal important information for people with T2D in future studies.

Pharmacological approaches for treating insomnia have potentially serious side effects on health. Several studies have shown an association between sleeping pill prescriptions and mortality in different populations [39-44]. Different sleep medications were associated with increased risk of fall [45], motor vehicle accidents [46], and suicidality [47]. Individuals with insomnia who use benzodiazepines or nonbenzodiazepines are at high risk of developing T2D because of potential changes in insulin secretion and sensitivity [48,49]. It is a widely held view that sleep apnea is a prevalent sleep disorder in people with T2D [50]. A possible explanation of increasing the severity of sleep apnea is that hypnotics are respiratory suppressants that might contribute in vital health issues for this population [51]. The insulin sensitivity improved in people with severe sleep apnea after receiving sleep hygiene, dietary counseling, and continuous passive airway pressure (CPAP) support, which suggests that the metabolic function in people with T2D might be improved by a sleep promotion program [52]. Thus, it is important to identify safe and effective nonpharmacological treatments for people with T2D and insomnia symptoms.

The American Academy of Sleep Medicine recommends cognitive behavioral therapy for insomnia (CBT-I) as the first line of treatment for people with insomnia [53]. A meta-analysis has shown CBT-I to produce clinically meaningful improvements in sleep outcomes including sleep latency (SL), sleep efficiency (SE), number of awakenings, and total sleep time (TST) [54]. In addition, CBT-I is designed to change sleep habits as well as address misconceptions about sleep and insomnia [55]. CBT-I is superior to sleep medications in terms of cost and long-term benefits [55]. Although there is currently limited evidence about the effect of CBT-I on people with T2D, CBT-I is a potentially effective intervention given insomnia’s relationship with glucose metabolism. We anticipate that CBT-I components will disrupt the associated physiological mechanisms between insomnia and T2D. Sleep restriction and stimulus control therapies are helpful in strengthening sleep homeostasis [56], which is also associated with the glucose regulation [57]. In adults with sleep restriction, increasing the TST with a simple low-cost intervention was associated with improvements in fasting insulin sensitivity [58]. Relaxation techniques are designed to minimize stress [59], which has a negative impact on the HPA axis in people with T2D [60]. These techniques are important additions in the treatment plan because of the high prevalence of psychological disorders such as depression and anxiety in people with T2D and insomnia [61]. The evidence has shown that sleep hygiene is not effective as monotherapy [62]. However, several items in the sleep hygiene could trigger DSCB, such physical activity, water consumption, and food schedule [30]. For example, avoiding excessive drinks at a night might help people with T2D minimize the bathroom visits after sleep onset [63]. The presence of nocturia is commonly reported in people with T2D, which could be one of the leading symptoms of insomnia [64]. CBT-I could compress the fragmentation of sleep, which may eventually help in reducing nocturia [64].

### Objectives and Hypotheses

The primary objective of this study is to establish a protocol for a pilot study to (1) investigate the effect of 6 sessions of CBT-I on insomnia severity in people with T2D and insomnia symptoms and (2) explore the effect of 6 sessions of CBT-I on sleep variability; fatigue; A_1c_; DSCB; sleep quality; daytime sleepiness; and the severity of depression, anxiety, and pain in people with T2D and insomnia symptoms. We hypothesized that people in the CBT-I group will have greater improvement in insomnia severity, sleep variability, fatigue, A_1c_, DSCB, sleep quality, daytime sleepiness, and severity of depression, anxiety, and pain compared with people receiving only health education (HE). We anticipate the improvement in insomnia severity will positively impact people with T2D and health outcomes because of the relationship between insomnia symptoms and diabetes-related health outcomes.

## Methods

### Trial Design

The study design will be a pilot randomized controlled trial (RCT). This study will have an allocation ratio of 1:1, and this pilot RCT will be using a superiority framework to test the effectiveness of the experimental CBT-I intervention. This protocol is in accord with the Standard Protocol Items: Recommendations for Interventional Trials 2013 statement [65], and the intervention will be described according to the Consolidated Standards of Reporting Trials 2010 guideline [66].

### Participants, Interventions, and Outcomes

#### Study Setting

This study will be conducted at the University of Kansas Medical Center (KUMC) in the United States. The study sites are also listed on ClinicalTrials.gov [67].

#### Eligibility Criteria

The inclusion and exclusion criteria are shown in Textboxes 1 and 2.

**Inclusion criteria.**

- Aged between 40 and 75 years.
- Have a type 2 diabetes diagnosis.
- Have a score of >10 on Insomnia Severity Index that indicates clinical insomnia—in addition, we will ask for reported symptoms of difficulty falling asleep, maintaining sleep, or waking up too ‎early at least three nights/week for the past 3 months.
- Are able to understand and follow verbal commands in English—the intervention and ‎questionnaires are available in English only; therefore, the participants must understand English language.
- Are able to travel to the University of Kansas Medical Center to attend all assessment and intervention ‎visits at ‎the Health Exercise and Aging Lab.

**Exclusion criteria.**

- Self-reported neurological diseases (eg, Alzheimer disease, Parkinson disease, traumatic brain injury, stroke, and multiple sclerosis)
- A score >4 on Stop-Bang questionnaire
- Failure to pass Restless Leg Syndrome Diagnostic Index
- Brief Pain Inventory score ≥7
- Beck Depression Scale score ≥21
- Generalized Anxiety Disorder–7 score ≥15
- Pregnant women
- Self-reported the following medical issues: chronic fatigue syndrome, fibromyalgia, bipolar, seizure disorders, and rheumatic diseases
- Speech deficits or significant auditory impairment
- Current night shift work
- Heavy alcohol drinker (≥15 drinks per week for men and ≥8 for women)
- Dialysis, blindness, or transfemoral amputation

In addition, during the phone screening, we will exclude the following people: 1) Those with scores >4 on Stop Bang items including snoring, tiredness, observed apnea, blood pressure, body mass index, age, neck circumference, and gender. People with sleep apnea symptoms commonly report poor sleep quality and insomnia [68]. The Snoring, Tiredness, Observed apnea, Blood pressure, Body mass index, Age, Neck circumference, and Gender (STOP Bang) questionnaire will be used to screen the common symptoms related to the high risk of sleep apnea, such as the presence of snoring behavior, wake time sleepiness or fatigue, and history of obesity or hypertension [69]. Neck circumference will be measured in the active screening session. STOP Bang showed higher sensitivity and specificity (93% and 28%, respectively), compared with other screening questionnaires at polysomnography-derived apnea-hypopnea index (score of 15), which indicates severe sleep apnea [70]. In addition, a meta-analysis [71] recommended using the STOP-Bang questionnaire as a screening for sleep apnea. If interested subjects have positive scores for 5 or more categories, they are classified as being at high risk of sleep apnea [69]. Those subjects will be excluded and recommended to visit their sleep specialists. We expect some people are diagnosed with sleep apnea and they are adhered with their CPAP machine. Those people will be still included by asking them to answer the STOP Bang questionnaire with considering CPAP utilization; 2) those failing to pass the RLS Diagnostic Index [72]. The RLS Diagnostic Index is based on an algorithm to give yes or no conclusion regarding the presence of RLS. The RLS Diagnostic Index includes questions about the urge to move legs or arms to detect the risk of RLS symptoms [72]. If the RLS Diagnostic Index indicates higher RLS risk, individuals *fail* the RLS Diagnostic Index, as RLS has a negative impact on individuals’ sleep, specifically, insomnia [73]; 3) those who are pregnant. As pregnancy impacts sleep, and insomnia is one of the major problems experienced in pregnancy, individuals who are pregnant will be excluded to reduce potential confounding factors [74]; 4) those who are heavy alcohol drinkers. In accordance with definitions established by the National Institute of Alcohol Abuse and Alcoholism [75], heavy drinking is typically defined as consuming 15 drinks or more per week for men and 8 drinks or more per week for women. Heavy alcohol consumption has been shown to be associated with sleep complaints among adults [76]. In addition, heavy drinking has been shown to be associated with subsequent insomnia symptoms in adults aged between 40 and 60 years [76]. Therefore, heavy alcohol drinkers will be excluded to reduce potential confounding factors; 5) those having any of the following self-reported problems: Neurological diseases—people with previous neurological disorders (eg, multiple sclerosis, Alzheimer disease, Parkinson disease, traumatic brain injury, and stroke—people with these neurological disorders usually report sleep problems [77-81]. Therefore, we want to focus on the interaction between insomnia and T2D; bipolar and seizure disorders—people with bipolar disorder and seizure disorder have complex sleep problems other than insomnia [82,83]. Furthermore, CBT-I is contraindicated for these populations [84]; chronic fatigue syndrome, fibromyalgia, and rheumatic diseases—people chronic fatigue syndrome is a medically unexplained disabling illness with nonrestorative sleep and potentially extended sleep duration [85]. Pain, fatigue, and poor sleep quality are common symptoms in people with fibromyalgia and rheumatic diseases [86-90]. Therefore, people with chronic fatigue syndrome, fibromyalgia, or rheumatic diseases will be excluded in this study; and dialysis, blindness, and transfemoral amputation—these diabetes complications may restrict people with T2D from performing components related to CBT-I; and 6) those who are shift workers. Shift workers usually report more physical and psychological distress, insomnia, and stress than non–shift workers [91]. In addition, CBT-I is contraindicated for people with shift work because CBT-I might increase sleepiness, which could put the individual at increased risk of harm.

During active screening session, the following interested participants will be excluded**:** 1) Those having scores ≥7 out of 10 on the Brief Pain Inventory (BPI) [92]. A subject with a score ≥7 out of 10 indicates severe pain symptoms. Diabetic patients with severe pain report high symptoms level of anxiety and depression and poor sleep quality [93]; 2) those having scores ≥21 ‎on the Beck Depression Inventory (BDI). BDI contains 21-item self-report inventory measuring the severity of depression symptoms in adolescents and adults, and participants with scores ≥21 indicate severe depression symptoms because scores above that point suggest severe symptoms level of depression [94]. Depression may lead to insomnia [95], and CBT-I may be a contraindication [96] or lead to contradictory results [97] for people with severe depression. Therefore, there is a need to exclude those people with severe symptoms level of depression from this study; 3) those having scores ≥15 on the Generalized Anxiety Disorder–7 (GAD-7) scale. Subjects who score ≥15 on GAD-7 indicate severe symptoms level of anxiety [98]. CBT-I is contraindicated for people with significant anxiety symptoms [96]; and 4) those having significant uncorrected visual, auditory impairment, and speech deficits. These health problems may affect the CBT-I delivery.

In addition, during the active screening session, we will confirm the ages between 40 and 75 years by obtaining the date of birth. People with diabetes aged between 40 and 75 years usually present with chronic insomnia [8,99]. A T2D diagnosis will be confirmed by each participant’s self-report. A study previously showed that the specificity of the prevalence and incidence of self-reported T2D was 84% and 97%, respectively, and sensitivity was 55% and 80%, respectively, compared with fasting glucose, A_1c_, and/or medication use [100]. In addition, a study suggested that self-reporting of T2D was sufficiently accurate [101]. In addition to the self-report of T2D diagnosis, we will also review the medication list to confirm the diagnosis during the screening active session.

#### Interventions: Experimental and Health Education

**Figure 1:**
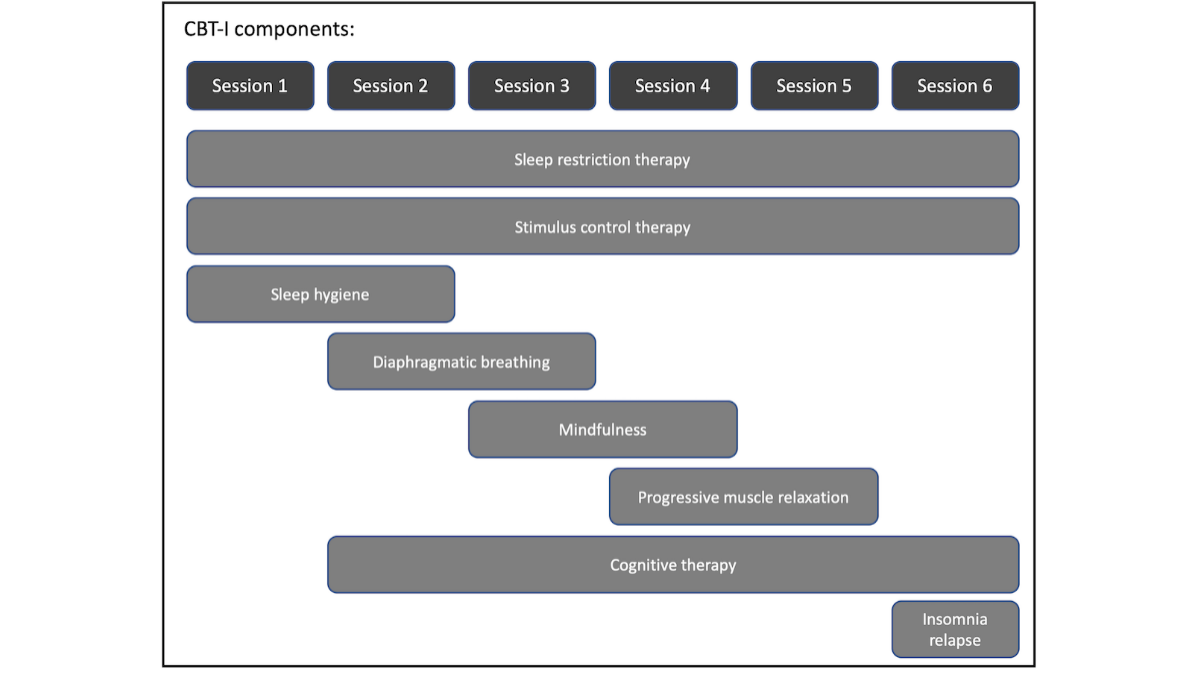
The timeline of the CBT-I (cognitive behavioral therapy for insomnia) intervention.

**Figure 2:**
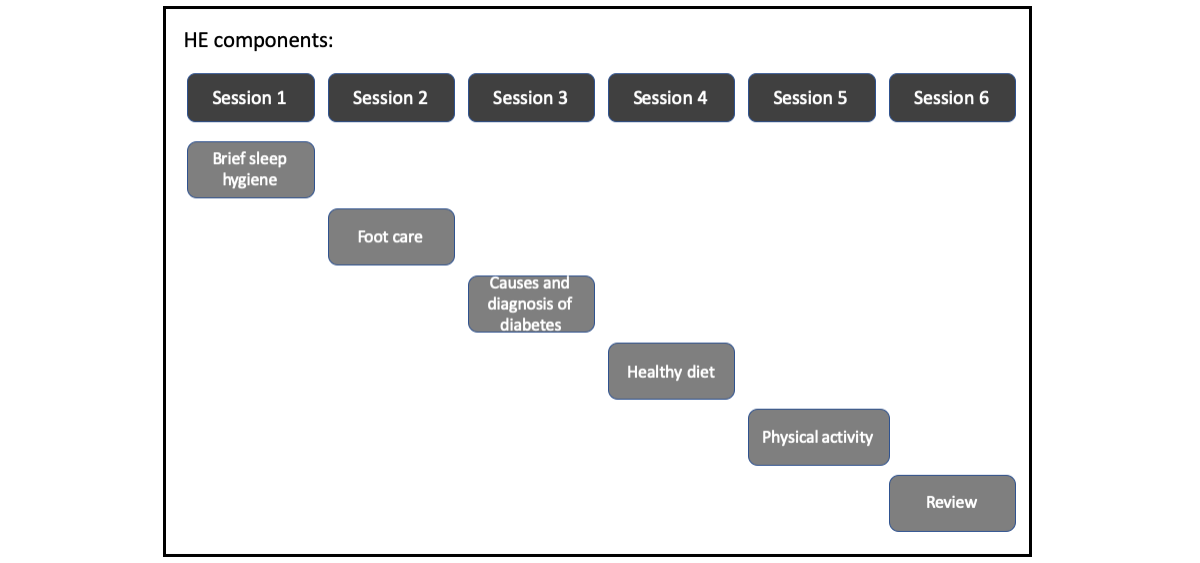
The timeline of Health Education.

All intervention sessions will be delivered by a trained CBT-I provider. The CBT-I provider is a physical therapist who completed coursework and a Mini-Fellowship in Behavioral Sleep Medicine through the University of Pennsylvania. Ongoing mentorship will be provided by an experienced CBT-I provider. All participants will receive 6 sessions over the course of 6 weeks of either CBT-I or HE (ie, 1 session per week for 6 weeks). Sessions will last for 1 hour for both groups to mitigate the impact of social interaction. We chose HE sessions as usual care for people with T2D. Textboxes 3 and 4 describe each intervention arm with all components. The timeline of each component for the CBT-I and HE groups is provided in Figures 1 and 2.

**Description of cognitive behavioral therapy for insomnia components.**

- Sleep restriction therapy
  - Time in bed will be limited to the total sleep time by identifying the wake time and total sleep time to increase the sleep efficiency. We will not prescribe the total time in bed to be less than 6 hours.
- Stimulus control therapy
  - This component strengthens the association between the bedtime and sleep only. We will ask participants to use the bed for only sleep and sexual activity to help train the brain. Participants will be asked to leave the bedroom if unable to fall asleep within 20 min and return when sleepy.
- Sleep hygiene
  - This component will minimize the influence of negative behaviors on sleep quality and quantity. The principles and the effects of diet, exercise, caffeine, alcohol, and environment on sleep behavior will be provided.
- Relaxation techniques
  - Diaphragmatic breathing technique promotes relaxation by using the diaphragm correctly while breathing.
  - Mindfulness reduces cognitive and somatic arousal. The principles of mindfulness (nonjudging, patience, trust, acceptance, and letting go) will be discussed.
  - Progressive muscle relaxation positively influences physiologically measured muscle tension.
  - Cognitive therapy changes detrimental beliefs and attitudes about sleep. We will work on reducing sleep effort, catastrophic predictions, worry about sleep, and fearing of insomnia relapse.
- Insomnia relapse
  - This component facilitates the understanding of the risk factors of reoccurrence. We will discuss the approaches to maintain clinical gains.

**Description of health education components.**

- Brief sleep hygiene
  - We will discuss 8 items of sleep hygiene including exercise, comfortable bedroom, temperature of bedroom, food, liquids, caffeine, alcohol consumption, smoking, and naps. Parts of sleep hygiene, such as consistent sleep schedule and association of bed with sleep, will not be included in this brief sleep hygiene education.
- Foot care education
  - We will provide foot care education regarding the demographic and comorbidity, foot pathology and assessment, and preventive interventions. In addition, we will provide the American Diabetes Association recommendation regarding foot hygiene.
- Causes and diagnosis of diabetes
  - We will provide information about diagnosis and classification of diabetes mellitus from the American Diabetes Association. The following topics will be discussed:
    - The definition and description of Diabetes Mellitus, classification of diabetes mellitus, and other categories of glucose regulation
    - Categories of increased risk for diabetes
    - Diagnostic criteria for diabetes mellitus
      - A short animation will be provided to explain how diabetes affects the body
- Healthy diet education
  - Different dietary approaches to manage type 2 diabetes will be discussed. Articles from American Diabetes Association website will be navigated).
- Physical activity education
  - We will use a guide for adults based on the 2008 Physical Activity Guidelines for Americans. We will discuss following points: *wondering about how much activity you need each week, want to be physically active but not sure where to begin, and started a program and would like tips on how to keep it up*.

##### Experimental Intervention

Participants allocated to the CBT-I group will meet with the CBT-I provider weekly for 1 hour of CBT-I sessions. CBT-I is designed to address cognitive and behavioral factors that perpetuate insomnia [102]. It includes several therapeutic components including sleep restriction therapy, stimulus control therapy, sleep hygiene, relaxation techniques, and cognitive therapy. At each session, the CBT-I provider will ask about any new difficulties, explain the outline of the session, calculate the SE of the previous 7 nights of sleep, and close the session with assessing any concerns and providing a new sleep diary. At each session, prescribed time in bed and out of bed will be determined based on calculation for SE of the weekly sleep diary. SE will be calculated as the ratio of TST and total bed time multiplied by 100. At each session, if the SE is greater than 90%, participants will be given the opportunity to go to bed 15 min earlier. If SE is between 85% and 89.9%, participants will be asked to remain on the same sleep schedule as currently prescribed. If it is less than 85%, they will be asked to move their bedtime 15 min later, although total time in bed will not be less than 6 hours.

This protocol intervention was designed based on a session-by-session guide [103].

###### Session 1 (60-90 Min)

Sleep restriction therapy, stimulus control therapy, and sleep hygiene will be started in this session. The sleep diary from the previous week will be reviewed to calculate the average SE from the previous 7 nights. In this session, subjects will learn the rationale and efficacy of using sleep restriction therapy and stimulus control therapy as a first line of treatment. Sleep restriction therapy is designed for individuals who are not able to initiate and/or maintain sleep [103,104]. This technique limits the time in bed to be equivalent to the TST by identifying the wake time and TST to increase the SE. Stimulus control promotes sleep drive and reinforces circadian entrainment by associating the bed to sleep only or for sex [103,104]. By applying the sleep restriction and stimulus control interventions, we will set prescribed time in bed and prescribed time out of bed by using the average of TST from previous week sleep diary and preferred time to wake up in the morning. Thus, the goal of this session is to align sleep with the opportunity to sleep, make plan in staying awake until the prescribed time in bed for sleep restriction therapy, and provide a list of activities during awake time or WASO for stimulus control therapy. In addition, sleep hygiene is implemented to minimize the influence of negative behaviors on sleep quality and quantity [103,104]. Sleep hygiene focuses on the impacts that diet, exercise, caffeine, alcohol, and environment can have on sleep [103,104].

###### Session 2 (30-60 Min)

Sleep titration, reviewing sleep hygiene, and introducing diaphragmatic breathing technique will be covered in this session. In this week, we will again review the sleep diary to confirm any necessary sleep titration (that is sleep restriction therapy adjustment). Sleep titration will be determined by measuring the individual’s SE. An SE >90% indicates positive gain that directs upward sleep opportunity. An SE between 85% and 90% score indicates marginal gain that maintains the sleep schedule. An SE <85% score indicates negative gain that directs downward sleep opportunity. If compliance issues arise during the sleep diary review, this session will focus on reinforcing the importance of sleep restriction and stimulus control therapies. Stress and anxiety symptoms are commonly reported in people with insomnia [105]. Thus, it is also important to implement relaxation techniques at the first sessions. We will emphasis relaxation therapy for people who are not able to relax because of varied of stressors or being an anxious. One of the relaxation therapies is the diaphragmatic breathing technique that promotes muscle relaxation, breathing performance, and memory relaxation. A brief diaphragmatic breathing handout and video will be utilized during the session.

###### Session 3 (30-60 Min)

Similar to sessions 1 and 2, the sleep diary will be reviewed for sleep titration, and we will introduce mindfulness. Upward or downward sleep titration will be determined based on the sleep diary. Mindfulness has shown positive effects in reducing cognitive and somatic arousal when combined with CBT-I for people with insomnia [106]. The principles of mindfulness (nonjudging, patience, trust, acceptance, and letting go) and its practice will also be introduced during this session.

###### Session 4 (30-60 Min)

Sleep titration and progressive muscle relaxation will be delivered in this session. Upward or downward sleep titration will be determined based on the sleep diary. Muscle relaxation therapy is a physiological intervention designed to measure and reduce muscle tension [107]. In addition, muscle relaxation therapy has been incorporated with CBT-I to improve insomnia and depression symptoms [107]. A brief progressive muscle relaxation handout and video will be utilized during the session.

###### Session 5 (30-60 Min)

Sleep titration and cognitive therapy will be delivered in this session. Cognitive therapy is designed to change detrimental beliefs and attitudes about sleep. The intervention content provided in sessions 1, 2, 3, or 4 may be similar to the cognitive therapy provided in session 5, although session 5 will focus on providing the cognitive therapy intervention in its entirety. During this session, we will work on reducing sleep effort, catastrophizing, anxiety about sleep, and insomnia relapse. Also, we will work on correcting negative sleep beliefs, particularly regarding insomnia. In addition, we will work on enhancing individuals’ willingness to modify the sleep-related behaviors and engage in good strategies. Finally, we will continue working on sleep titration to optimize the SE.

###### Session 6 (30 Min)

Assessing global treatment gains and relapse prevention education will be the focus in this session. We will review the SE of each session to graphically demonstrate the participant’s SE over the course of this intervention. This process will help in providing information that facilitates chronic insomnia and understands the risk factors of reoccurrence. Finally, we will discuss the approaches to maintain clinical gains and fix insomnia returns, and we will schedule participants for the reassessment session.

During each session, the CBT-I provider will use 2 documentation sheets that are nonspecific to CBT-I: a checklist and tracking sheet. These sheets will help the CBT-I provider for quality assurance and standardization of treatment sessions across participants. We do not expect these sheets to contribute to the intervention or the outcomes of this study.

The participants will be called 1 day before each session to confirm their session appointment the following day and remind them to bring their completed sleep diary. In addition, a folder will be provided at the first session to keep provided materials together for review. The CBT-I sessions will be audio recorded to assess treatment integrity if the subject agrees.

CBT-I intervention fidelity will be assessed by an independent CBT-I expert who will use a scoring sheet to assess CBT-I provider’s compliance in utilizing the manual to deliver the CBT-I. The CBT-I provider will be scored on 5 scales from 0 (poor) to 6 (excellent) based on (1) how they address immediate concern, (2) how they explain the outline of the session, (3) how they discuss the sleep diary outcomes, (4) their adherence in providing the intervention, and (5) their competency in delivering each session.

##### Control Group

Participants allocated to the HE group will meet with the CBT-I provider weekly for 1 hour of HE sessions. The HE sessions include several components including brief sleep hygiene, foot care, diabetes classifications, healthy diet, and physical activity. During all sessions, subjects will be encouraged to engage in discussion through open questions about their experience of diabetes and lifestyle as well as their comprehension of the provided materials. Similar to the CBT-I group, session tracking sheets will be used to track new difficulties or concerns and provided education.

#### Outcomes

##### Demographic and Clinical Variables

Age, race, ethnicity, sex, marital status, education, employment, diabetes duration, medication list, and body mass index will be gathered at the first assessment session.

##### Sleep Outcomes

###### Insomnia Severity

The Insomnia Severity Index (ISI) is a self-report measure designed to evaluate the nature, severity, and impact of insomnia [108]. The ISI is a valid and reliable measure of clinical insomnia and involves 7 questions, each rated on a 0 to 4 Likert scale. Total scores range from 0 to 28, with higher scores indicating greater insomnia severity [108]. The internal consistency of ISI was excellent for community sample and clinical sample (alpha=.90 and alpha=.91, respectively). The cutoff score>10 on the ISI provided optimal sensitivity and specificity for the detection of insomnia based on the Diagnostic and Statistical Manual of Mental Disorders, fifth edition, diagnostic criteria (area under the curve=0.82; 95% confidence interval 0.78-0.86) [109].

###### Sleep Variability

The Actigraph device is a small, noninvasive device worn on the nondominant wrist that records limb movements using electrical impulses, and it has been validated for use in people with insomnia [110]. Sleep parameters including SE, SL, TST, and WASO will be measured. In addition to the Actigraph, we will also use a sleep diary to allow for better estimation of the time in and out of bed as well as for removing invalid sleep periods that are measured by the Actigraph [111]. The sleep diary will also measure total time spent in bed, total time spent out of bed, number of awakenings, number of bathroom visits, and glucose level before and after sleep time. All sleep parameters will be presented in averages of 7 nights and the coefficient of variance (CV) will be calculated using the following equation (CV=standard deviation/mean×100) for each objective and subjective sleep parameters—SE, SL, TST, and WASO—to analyze objectively within-subject variability of nighttime sleep of 7 nights. This calculation will provide a percentage value with a higher number suggesting higher variability [112].

###### Daytime Sleepiness

The Epworth Sleepiness Scale (ESS) uses 8 items on a 4-point Likert scale, where the subjects rate how likely they would be to fall asleep in 8 different states of daily activities. The ESS has demonstrated satisfactory psychometric properties such as test-retest reliability (r=.82) and internal consistency (alpha=.88). The cutoff point is ≥10 to distinguish between normal from pathological sleepiness [24].

###### Sleep Quality

The Pittsburgh Sleep Quality Index (PSQI) is a validated 19-item questionnaire that differentiates between poor and good sleepers. The PSQI uses 7 items on a 4-point Likert scale, and it yields a global sleep quality score that ranges from 0 to 21. Poor sleepers have scores >5, with this cutoff global PSQI score providing satisfactory sensitivity (89.6%) and specificity (86.5%). In our study, we will use a 3-factor scoring model for the PSQI (SE, perceived sleep quality, and daily disturbances), which has been tested and validated [113]. Sleep duration and SE were classified under the SE factor; subjective sleep quality, SL, and the use of sleeping medications were categorized under the perceived sleep quality factor; and the frequency of sleep disturbances and daytime dysfunction were classified under the daily disturbances factor.

##### Diabetes Outcomes

###### Diabetes Self-Care Behavior

The diabetic care profile (DCP) uses items on 5-point Likert scales to evaluate the frequency of symptoms related to diabetes. The DCP is a validated instrument that measures self-reported diabetes control and psychological and social factors associated with the management of diabetes [114,115].

###### Glycemic Control

A_1c_ will be determined using the hemoglobin A_1c_ test by a disposable blood finger stick test using (A1cNow+ kit; TMS Company). The A_1c_ indicates the average blood glucose level of people with diabetes over the previous 2 to 3 months and represents the current management of diabetes [116]. Every 1% drop in A_1c_ is associated with improved outcomes with no threshold effect [117].

###### Glucose Level

Random glucose levels will be measured using glucose meter (Contour Next EZ Blood Glucose Monitoring System, Model 7252). The results of glucose level will be presented in milligrams per deciliter to document nonfasting glucose levels [118].

##### Health Outcomes

###### Fatigue Severity

Fatigue symptoms will be measured using the Fatigue Severity Scale (FSS), which is a 9-item questionnaire that has been validated in people with diabetes [119]. The FSS measures fatigue across 5 subscales including motivation, exercise, interference with work, family, or social life. These subscales have total scores where a score <4 indicates no fatigue, scores between 4 and 4.9 indicate moderate fatigue, and a score >5 indicates severe fatigue [119].

###### Pain Severity Symptoms

The BPI is a valid and reliable measure to assess painful diabetic peripheral neuropathy [120]. We measured the means of the severity scale and the interference scale of the BPI.

###### Depression Symptoms

The BDI has high reliability and good validity [121,122]. It contains 21 self-reported items on a 3-point Likert scale, with scores ≥21 indicating severe depression symptoms [121,122].

###### Anxiety Symptoms

The GAD-7 uses 7 items on a 3-point Likert scale. The total score of the GAD-7 ranges from 0 to 21, with higher scores indicating severe anxiety symptoms. It has been shown to be highly sensitive and specific for the detection of anxiety symptoms, and it is correlated with other anxiety scales [123].

### Participant Timeline

**Figure 3:**
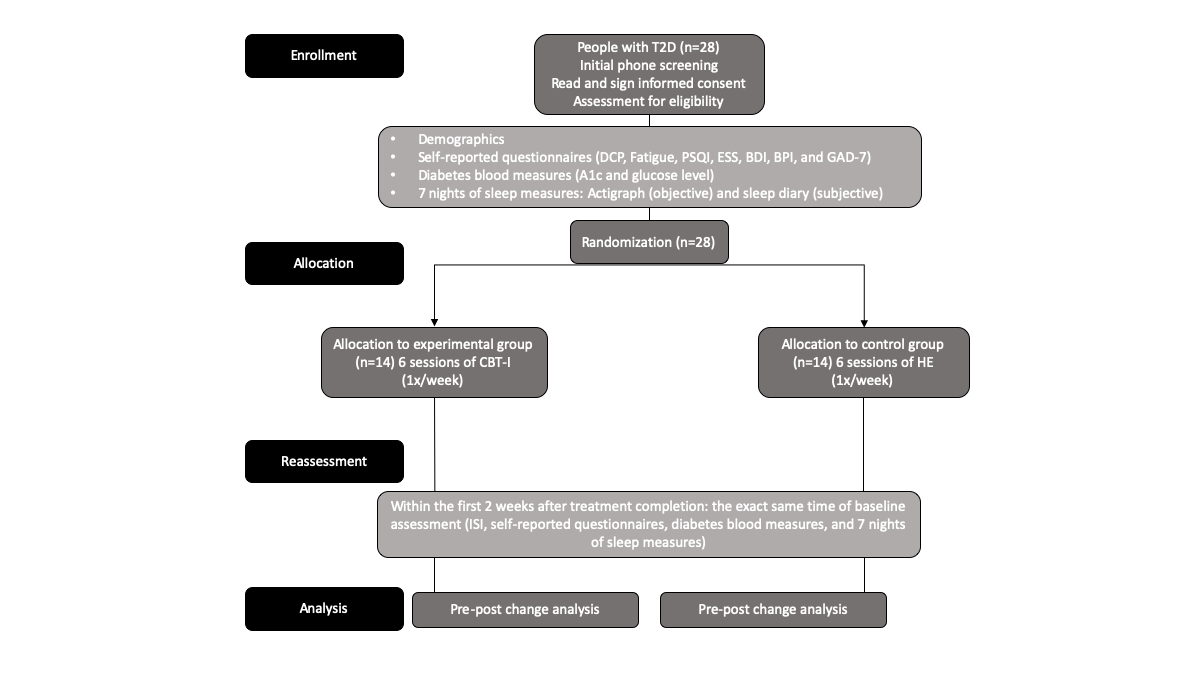
Consort of the project. A_1c_: glycemic control; BDI: Beck Depression Inventory; BPI: Brief Pain Inventory; CBT-I: cognitive behavioral therapy for insomnia; DCP: diabetic care profile DSCB: diabetes self-care behavior; ESS: Epworth Sleepiness Scale; GAD-7: Generalized Anxiety Disorder–7; ISI: Insomnia Severity Index; PSQI: Pittsburgh Sleep Quality Index; and T2D: type 2 diabetes.

All measurements will be performed at baseline and 1 week after treatment completion (Figure 3). Participants who wish to withdraw during the intervention will be asked to complete the reassessment session.

At initial contact with a potential subject, a phone screening or diabetes clinic interview will be conducted by a member of the research team to determine whether an individual qualifies to progress to an in-person screening for the study. The phone screening interview assesses participant eligibility according to age, self-report of T2D diagnosis, insomnia severity, ability to understand English, STOP-Bang score, RLS Diagnostic Index, pregnancy status, alcohol use, night-shift work, and undiagnosed neurological disorders.

Individuals passing the phone screening will be scheduled for an in-person screening session to assess eligibility according to symptoms of pain, depression, and anxiety.

Subjects will undergo the consent process in a private room at KUMC before completing any of the in-person screening assessments. Individuals passing the in-person screening will then immediately begin baseline assessment.

#### Sample Size

To detect the effect of CBT-I on people with T2D and symptoms of insomnia, the change in pre-post ISI was used to determine sample size. Pre-post changes using the minimal clinically meaningful difference of 8 points for the ISI in a previous study [124] were used to estimate the effect size. This calculation resulted in 10 participants per group to reject the null hypothesis of equal means when the population mean difference equals 8 with a standard deviation of 7. We accounted for an expected attrition rate of 40%, which indicated 28 subjects in both groups to detect the significant difference between groups after allowing for attrition at a .05 significant level and power of .80.

#### Recruitment

Subjects will be recruited from diabetes and sleep clinics at KUMC, university advertisements, community centers in Kansas City, flyers, personal referrals and newsletters, and a registry of patients from KUMC who have signed up to be contacted about potential research opportunities.

### Assignment of Interventions (for Controlled Trials)

#### Allocation Sequence Generation

We will use random mixed block size randomization [125] to assign participants to either CBT-I (n=14) or HE (n=14) groups. Participants will be stratified by age where 62 years is the value that will stratify participants into either the older (63-75 years) or the younger (40-62 years) age group. The reason we chose age as a blocking variable is that the impact of age on sleep is more pronounced than gender [126], as older adults often have poorer [127] and lower slow wave [128] sleep, as compared with young adults.

#### Allocation Concealment Mechanism

Participant allocations will be placed in sealed envelopes. The envelopes are prepared by a research assistant, who withholds this information from the CBT-I provider. After finishing the baseline assessment, participants will be asked to open the sealed envelope to disclose their group allocation. Microsoft Excel will be used to create the randomization lists.

#### Allocation Implementation

A computer will be used to generate the random mixed block size randomization sequences. Results of the generator will be concealed from the assessor and CBT-I provider. Participants will be asked to open the sealed enveloped after informed consent and baseline assessment are completed.

#### Blinding

The assessor, who is blinded to group allocation, will score the Actigraph data. The assessor will have experience in scoring criteria and no involvement in providing the interventions. The CBT-I provider will not be blinded in this study.

#### Data Collection, Management, and Analysis

##### Data Collection and Methods

Insomnia severity (primary outcome); sleep variability; fatigue; DSCB; A_1c_; daytime sleepiness; sleep quality; glucose levels; and symptoms of depression, anxiety, and pain will be measured a week before and after the intervention.

##### Data Management

All study-related procedure will be performed at Georgia Holland laboratory in Hemenway Life Sciences Innovation Center on the KUMC campus. All obtained participant records will be kept in locked cabinet inside the Georgia Holland laboratory. Electronic study data will be saved in the KUMC Research Electronic Data Capture system. For voice records, all tapes will be saved on a secure university-supported network drive.

##### Statistical Methods

A chi-square test will be used to compare between-group differences in categorical variables. Independent 2-sample *t* tests will be used to compare differences in continuous between-group demographic characteristics and clinical variables.

For the main analysis**,** the effect of the CBT-I intervention will be investigated by calculating change scores for the insomnia severity; DSCB; A_1c_; fatigue; sleep quality; daytime sleepiness; and symptoms of depression, anxiety, and pain. In addition, for variability of each sleep outcomes (SE, SL, TST, and WASO), CV of 7 nights before the pre- and postintervention assessments will be calculated, and change scores will be calculated. Then, independent sample *t* tests will be utilized to investigate the between-group difference in the change score means of all outcomes. A completer vs noncompleter analysis will be performed. For all analyses, alpha level will be set at .05.

Owing to the complex relationship between insomnia and T2D, some factors are needed to be controlled to fully investigate the relationship. Owing to the small sample size and possible covariates that might not be included in the power calculation, these complex relationships will be investigated using exploratory analyses. Post hoc analysis using the type and number of medications will used to address the potential confounding effects on the outcomes. Univariate linear regression will be used to control for demographic and clinical variables (covariates). The decision to perform these analyses with demographic and clinical variables will be made if there are significant between-group differences at baseline in depression symptoms, anxiety symptoms, pain symptoms, gender, diabetes duration, or body mass index. Mixed models will be used to account for the correlation between times in pre- and posttest (7 nights as random factor) sleep variability (as dependent variable) and facilitate adjustment for covariates to compare the difference in sleep variability between the CBT-I and HE groups (groups as fixed factor). Covariates will be determined if there is a difference in the baseline assessment for demographic and clinical outcomes. Those participants who are treated with CPAP will be asked to report their compliance using CPAP during baseline and postintervention assessments. Subjects who are using VPAP will be given a modified sleep diary to check off nights of CPAP compliance during the assessment sessions. An exploratory subanalysis will be utilized to investigate the difference in insomnia severity between compliance and noncompliance with CPAP. Noncompliance is defined as (1) missing more than 2 nights during the 7 nights period that the participant is wearing the Actigraph or (2) using the CPAP for <4 hours per night during this study.

##### Monitoring and Ethics

###### Data Monitoring

The primary investigator will review the dataset at least semiannually. The primary investigator’s evaluation will be focused on the quality of data collection and data management. In addition, the investigators will review data in an ongoing manner for accuracy, both at a time when these data are entered into the database and during analysis.

###### Harms

During the pre and postassessment sessions, testing will be stopped if the subjects show signs of low blood sugar (<70 mg/dL) or if signs of dizziness or headache are noted by the assessor or reported by the participant. During assessment sessions, participants also will be instructed to stop the test at any time for a rest break, as often as needed.

There is a risk of skin redness may be associated with wearing the Actigraph for 1 week. The risks of wearing the Actigraph are nearly the same as wearing a wrist watch. If skin redness or inflammation happened, subjects may remove the Actigraph and immediately report the symptoms to research personnel. In addition, there is a risk of minor electrical shock if the Actigraph is damaged. If damage to the Actigraph occurs, subject will be asked to return it to our lab, and they will be given a replacement.

Initially, participating in a CBT-I intervention may have an increase in sleepiness, which may impact participants’ fatigue, thinking ability, or functional abilities. It is anticipated that this increase in sleepiness will be temporary and should help participants sleep better in the long term.

During the in-person screening session, if suicidal intent is identified through either the BDI (question number 9, with a *2* or a *3*) or verbal statement from the participant, a suicidality protocol will be followed. The suicidality protocol is designed to provide the researcher with contact information for appropriate psychology and psychiatric professionals at KUMC.

####### Research Ethics Approval

The study will be performed in accordance with KUMC’s Institutional Review Board and Human Subjects Committee. No individuals will be excluded based on sex, race, or ethnicity. Interested participants will be administered a structured screening interview to determine their eligibility for the study. During the consent session, all interested participant will be informed about the study’s objective, risks, procedure, and potential benefits (or lack thereof).

######## Consent or Assent

Consent will be obtained in Georgia Holland Health Exercise and Aging Lab on the main campus of KUMC. Participants will be encouraged to ask any questions about the study as much as they need, and members of research study will answer their questions. In addition, participants will be informed if there is any change in the protocol to sign a new consent form.

######## Confidentiality

All data will be deidentified and stored on the KUMC research private drive, which will be secured and backed up every night. The working dataset will be stored on a password-protected computer in the primary investigator’s laboratory, with access restricted to study researchers who are actively working with these data. All subject files and documents will be stored in a locked cabinet.

## Results

A total of 28 participants with T2D and insomnia symptoms recruited from February 2019. This study currently completed the recruitment stage. The completion date for the study was September 2019. Our results will describe the changes in insomnia severity; sleep variability; fatigue; A_1c_; DSCB; and severity of depression, anxiety, and pain. We will report our results in tables and figures using SPSS and GraphPad, respectively.

## Discussion

### Overview

Our study will be the first in conducting an RCT using CBT-I for people with T2D. If this study indicates that 6 sessions of CBT-I are effective in improving sleep and diabetes outcomes in people with T2D and insomnia symptoms when compared with HE, CBT-I could be implemented as an effective and safe treatment for this population, although more research will be needed to verify the findings of this pilot RCT.

Pharmacological interventions for sleep difficulties have shown harmful effects on people with T2D. There is a need to better understand safe intervention benefits in people with T2D. This study will contribute to the management of T2D using behavioral sleep intervention as an effective and safe treatment for people with insomnia symptoms. The results will contribute to the literature by examining the effect of CBT-I on both sleep and diabetes outcomes. This will help in understanding the effectiveness of short duration intervention designed for people with insomnia symptoms.

### Strengths

The study strengths include utilizing important methods for people with T2D, such as objective measures, design, and safe intervention. Determining sleep variability using objective and subjective measures will accurately detect sleep improvement after an intervention. Using comparative groups to understand the effect of CBT-I on insomnia symptoms, sleep variability, fatigue, and diabetes-related health outcomes will add new information to the literature and improve the understanding of clinical conditions. Previous studies recommend optimizing the sleep quality and quantity for people with comorbidities. Understanding the effect of CBT-I in people with T2D will expand the generalizability of using this type of interventions.

### Limitations

Some limitations in this protocol might be important to consider in future studies. First, we will not confirm the diagnosis of T2D using the current American Diabetes Association guidelines. However, a study showed that the specificity of prevalent self-reported diabetes and incident self-reported diabetes were 84% and 97% and sensitivity of 55% and 80%, respectively, compared with fasting glucose, A_1c_, and/or medication use [100]. In addition, a study suggests that self-report of diabetes is sufficiently accurate [101]. To overcome this limitation, we will review the medication list to confirm T2D diagnosis during the in-person screening visit. Second, we might not able to distinguish the improvement in insomnia severity between controlled vs uncontrolled diabetes, which might be examined under future sleep behavioral therapy studies. Third, our study will be powered based on the ISI, and we recommend future studies to choose a diabetes outcome for conducting the power calculation. Fourth, the participants will not be blinded in the study, which may result in the control group participants looking for CBT-I providers outside of the study. We will monitor any change in pharmacological or nonpharmacological treatments during the postintervention session to help explain unexpected results. It is possible, however, that some participant may not reveal this information, which might influence the outcomes. Finally, we will not be able to monitor CPAP compliance during the intervention, but we will follow up with people in the CBT-I group to ensure no issue wearing a CPAP machine every session.

## Abbreviations

A_1c_: glycemic control
BDI: Beck Depression Inventory
BPI: Brief Pain Inventory
CBT-I: cognitive behavioral therapy for insomnia
HE: Health Education
CV: coefficient of variance
DCP: diabetic care profile
DSCB: diabetes self-care behavior
ESS: Epworth Sleepiness Scale
FSS: Fatigue Severity Scale
GAD-7: Generalized Anxiety Disorder–7
HPA: hypothalamic-pituitary-adrenal
ISI: Insomnia Severity Index
KUMC: University of Kansas Medical Center
CPAP: continuous passive airway pressure
PSQI: Pittsburgh Sleep Quality Index
RCT: randomized controlled trial
RLS: restless leg syndrome
SE: sleep efficiency
SL: sleep latency
STOP-Bang: snoring, tiredness, observed apnea, blood pressure, body mass index, age, neck circumference, and gender
T2D: type 2 diabetes
TNF: tumor necrosis factor
TST: total sleep time
WASO: waking after sleep onset

## References

[1] Strand LB, Carnethon M, Biggs ML, Djoussé L, Kaplan RC, Siscovick DS, Robbins JA, Redline S, Patel SR, Janszky I, Mukamal KJ (2015). Sleep disturbances and glucose metabolism in older adults: the cardiovascular health study. Diabetes Care.

[2] Sinclair A, Dunning T, Rodriguez-Mañas L (2015). Diabetes in older people: new insights and remaining challenges. Lancet Diabetes Endocrinol.

[3] American Diabetes Association (2019). 2. Classification and diagnosis of diabetes: standards of medical care in diabetes-2019. Diabetes Care.

[4] American Diabetes Association (2014). Diagnosis and classification of diabetes mellitus. Diabetes Care.

[5] Chauhan A, Sharma SD (2016). Comments on: Microvascular and macrovascular complications in diabetes mellitus: Distinct or continuum?. Indian J Endocrinol Metab.

[6] Surani S, Brito V, Surani A, Ghamande S (2015). Effect of diabetes mellitus on sleep quality. World J Diabetes.

[7] Buckley TM, Schatzberg AF (2005). On the interactions of the hypothalamic-pituitary-adrenal (HPA) axis and sleep: normal HPA axis activity and circadian rhythm, exemplary sleep disorders. J Clin Endocrinol Metab.

[8] Vgontzas AN, Liao D, Pejovic S, Calhoun S, Karataraki M, Bixler EO (2009). Insomnia with objective short sleep duration is associated with type 2 diabetes: A population-based study. Diabetes Care.

[9] Vgontzas AN, Liao D, Pejovic S, Calhoun S, Karataraki M, Basta M, Fernández-Mendoza J, Bixler EO (2010). Insomnia with short sleep duration and mortality: the Penn State cohort. Sleep.

[10] Balbo M, Leproult R, Van Cauter E (2010). Impact of sleep and its disturbances on hypothalamo-pituitary-adrenal axis activity. Int J Endocrinol.

[11] Chasens ER, Umlauf MG (2003). Nocturia: a problem that disrupts sleep and predicts obstructive sleep apnea. Geriatr Nurs.

[12] Umlauf MG, Chasens ER (2003). Sleep disordered breathing and nocturnal polyuria: nocturia and enuresis. Sleep Med Rev.

[13] Balbo M, Leproult R, Van Cauter E (2010). Impact of sleep and its disturbances on hypothalamo-pituitary-adrenal axis activity. Int J Endocrinol.

[14] Martins RC, Andersen ML, Tufik S (2008). The reciprocal interaction between sleep and type 2 diabetes mellitus: facts and perspectives. Braz J Med Biol Res.

[15] Budhiraja R, Roth T, Hudgel DW, Budhiraja P, Drake CL (2011). Prevalence and polysomnographic correlates of insomnia comorbid with medical disorders. Sleep.

[16] Chasens ER, Sereika SM, Burke LE, Strollo PJ, Korytkowski M (2014). Sleep, health-related quality of life, and functional outcomes in adults with diabetes. Appl Nurs Res.

[17] Skomro RP, Ludwig S, Salamon E, Kryger MH (2001). Sleep complaints and restless legs syndrome in adult type 2 diabetics. Sleep Med.

[18] Ahmad S, Gupta M, Gupta R, Dhyani M (2013). Prevalence and correlates of insomnia and obstructive sleep apnea in chronic kidney disease. N Am J Med Sci.

[19] Kamel NS, Gammack JK (2006). Insomnia in the elderly: cause, approach, and treatment. Am J Med.

[20] Hajak G, SINE Study Group. Study of Insomnia in Europe (2001). Epidemiology of severe insomnia and its consequences in Germany. Eur Arch Psychiatry Clin Neurosci.

[21] Sofi F, Cesari F, Casini A, Macchi C, Abbate R, Gensini GF (2014). Insomnia and risk of cardiovascular disease: a meta-analysis. Eur J Prev Cardiol.

[22] Laugsand LE, Vatten LJ, Platou C, Janszky I (2011). Insomnia and the risk of acute myocardial infarction: a population study. Circulation.

[23] Sivertsen B, Pallesen S, Glozier N, Bjorvatn B, Salo P, Tell GS, Ursin R, Øverland S (2014). Midlife insomnia and subsequent mortality: the Hordaland health study. BMC Public Health.

[24] Chasens ER, Korytkowski M, Sereika SM, Burke LE (2013). Effect of poor sleep quality and excessive daytime sleepiness on factors associated with diabetes self-management. Diabetes Educ.

[25] Williams GC, McGregor HA, Zeldman A, Freedman ZR, Deci EL (2004). Testing a self-determination theory process model for promoting glycemic control through diabetes self-management. Health Psychol.

[26] Pilcher JJ, Ginter DR, Sadowsky B (1997). Sleep quality versus sleep quantity: relationships between sleep and measures of health, well-being and sleepiness in college students. J Psychosom Res.

[27] Lemola S, Ledermann T, Friedman EM (2013). Variability of sleep duration is related to subjective sleep quality and subjective well-being: an actigraphy study. PLoS One.

[28] Weaver TE, Laizner AM, Evans LK, Maislin G, Chugh DK, Lyon K, Smith PL, Schwartz AR, Redline S, Pack AI, Dinges DF (1997). An instrument to measure functional status outcomes for disorders of excessive sleepiness. Sleep.

[29] Altevogt BM, Colten HR, Institute of Medicine, Board on Health Sciences Policy, Committee on Sleep Medicine and Research (2006). Sleep Disorders And Sleep Deprivation: An Unmet Public Health Problem.

[30] Luyster FS, Dunbar-Jacob J (2011). Sleep quality and quality of life in adults with type 2 diabetes. Diabetes Educ.

[31] Molzof HE, Emert SE, Tutek J, Mulla MM, Lichstein KL, Taylor DJ, Riedel BW (2018). Intraindividual sleep variability and its association with insomnia identity and poor sleep. Sleep Med.

[32] Patel SR, Hayes AL, Blackwell T, Evans DS, Ancoli-Israel S, Wing YK, Stone KL, Osteoporotic Fractures in Men (MrOS), Study of Osteoporotic Fractures (SOF) Research Groups (2014). The association between sleep patterns and obesity in older adults. Int J Obes (Lond).

[33] Luik AI, Zuurbier LA, Hofman A, van Someren EJ, Tiemeier H (2013). Stability and fragmentation of the activity rhythm across the sleep-wake cycle: the importance of age, lifestyle, and mental health. Chronobiol Int.

[34] Sprague AH, Khalil RA (2009). Inflammatory cytokines in vascular dysfunction and vascular disease. Biochem Pharmacol.

[35] Vgontzas AN, Bixler EO, Basta M (2010). Obesity and sleep: a bidirectional association?. Sleep.

[36] Taylor DJ, Lichstein KL, Durrence HH, Reidel BW, Bush AJ (2005). Epidemiology of insomnia, depression, and anxiety. Sleep.

[37] Aikens JE, Perkins DW, Lipton B, Piette JD (2009). Longitudinal analysis of depressive symptoms and glycemic control in type 2 diabetes. Diabetes Care.

[38] Spiegel K, Knutson K, Leproult R, Tasali E, van Cauter E (2005). Sleep loss: a novel risk factor for insulin resistance and Type 2 diabetes. J Appl Physiol (1985).

[39] Kripke DF, Garfinkel L, Wingard DL, Klauber MR, Marler MR (2002). Mortality associated with sleep duration and insomnia. Arch Gen Psychiatry.

[40] Kripke DF, Klauber MR, Wingard DL, Fell RL, Assmus JD, Garfinkel L (1998). Mortality hazard associated with prescription hypnotics. Biol Psychiatry.

[41] Charlson F, Degenhardt L, McLaren J, Hall W, Lynskey M (2009). A systematic review of research examining benzodiazepine-related mortality. Pharmacoepidemiol Drug Saf.

[42] Hublin C, Partinen M, Koskenvuo M, Kaprio J (2007). Sleep and mortality: a population-based 22-year follow-up study. Sleep.

[43] Weich S, Pearce HL, Croft P, Singh S, Crome I, Bashford J, Frisher M (2014). Effect of anxiolytic and hypnotic drug prescriptions on mortality hazards: retrospective cohort study. Br Med J.

[44] Sivertsen B, Madsen IE, Salo P, Tell GS, Øverland S (2015). Use of sleep medications and mortality: the Hordaland health study. Drugs Real World Outcomes.

[45] Ambrose AF, Paul G, Hausdorff JM (2013). Risk factors for falls among older adults: a review of the literature. Maturitas.

[46] Yang Y, Lai J, Lee C, Wang J, Chen P (2011). Increased risk of hospitalization related to motor vehicle accidents among people taking zolpidem: a case-crossover study. J Epidemiol.

[47] Carlsten A, Waern M (2009). Are sedatives and hypnotics associated with increased suicide risk of suicide in the elderly?. BMC Geriatr.

[48] Lin C, Yeh M, Harnod T, Lin C, Kao C (2015). Risk of type 2 diabetes in patients with nonapnea sleep disorders in using different types of hypnotics: a population-based retrospective cohort study. Medicine (Baltimore).

[49] Gramaglia E, Ramella Gigliardi V, Olivetti I, Tomelini M, Belcastro S, Calvi E, Dotta A, Ghigo E, Benso A, Broglio F (2014). Impact of short-term treatment with benzodiazepines and imidazopyridines on glucose metabolism in healthy subjects. J Endocrinol Invest.

[50] Wang X, Bi Y, Zhang Q, Pan F (2013). Obstructive sleep apnoea and the risk of type 2 diabetes: a meta-analysis of prospective cohort studies. Respirology.

[51] Kripke DF (2016). Hypnotic drug risks of mortality, infection, depression, and cancer: but lack of benefit. F1000Res.

[52] Weinstock TG, Wang X, Rueschman M, Ismail-Beigi F, Aylor J, Babineau DC, Mehra R, Redline S (2012). A controlled trial of CPAP therapy on metabolic control in individuals with impaired glucose tolerance and sleep apnea. Sleep.

[53] Morgenthaler T, Kramer M, Alessi C, Friedman L, Boehlecke B, Brown T, Coleman J, Kapur V, Lee-Chiong T, Owens J, Pancer J, Swick T, American Academy of Sleep Medicine (2006). Practice parameters for the psychological and behavioral treatment of insomnia: an update. An american academy of sleep medicine report. Sleep.

[54] Trauer JM, Qian MY, Doyle JS, Rajaratnam SM, Cunnington D (2015). cognitive behavioral therapy for chronic insomnia: a systematic review and meta-analysis. Ann Intern Med.

[55] Mitchell MD, Gehrman P, Perlis M, Umscheid CA (2012). Comparative effectiveness of cognitive behavioral therapy for insomnia: a systematic review. BMC Fam Pract.

[56] Akerstedt T, Kecklund G, Ingre M, Lekander M, Axelsson J (2009). Sleep homeostasis during repeated sleep restriction and recovery: support from EEG dynamics. Sleep.

[57] Taub LM, Redeker NS (2008). Sleep disorders, glucose regulation, and type 2 diabetes. Biol Res Nurs.

[58] Leproult R, Deliens G, Gilson M, Peigneux P (2015). Beneficial impact of sleep extension on fasting insulin sensitivity in adults with habitual sleep restriction. Sleep.

[59] Surwit RS, van Tilburg MA, Zucker N, McCaskill CC, Parekh P, Feinglos MN, Edwards CL, Williams P, Lane JD (2002). Stress management improves long-term glycemic control in type 2 diabetes. Diabetes Care.

[60] Rosmond R (2003). Stress induced disturbances of the HPA axis: a pathway to Type 2 diabetes?. Med Sci Monit.

[61] Golden SH, Shah N, Naqibuddin M, Payne JL, Hill-Briggs F, Wand GS, Wang N, Langan S, Lyketsos C (2017). The prevalence and specificity of depression diagnosis in a clinic-based population of adults with type 2 diabetes mellitus. Psychosomatics.

[62] Schoicket SL, Bertelson AD, Lacks P (1988). Is sleep hygiene a sufficient treatment for sleep-maintenance insomnia?. Behav Ther.

[63] Mobley DF, Baum N (2014). Etiology, evaluation, and management of nocturia in elderly men and women. Postgrad Med.

[64] Tyagi S, Resnick NM, Perera S, Monk TH, Hall MH, Buysse DJ (2014). Behavioral treatment of insomnia: also effective for nocturia. J Am Geriatr Soc.

[65] Chan A, Tetzlaff JM, Altman DG, Laupacis A, Gøtzsche PC, Krleža-Jerić K, Hróbjartsson A, Mann H, Dickersin K, Berlin JA, Doré CJ, Parulekar WR, Summerskill WS, Groves T, Schulz KF, Sox HC, Rockhold FW, Rennie D, Moher D (2013). SPIRIT 2013 statement: defining standard protocol items for clinical trials. Ann Intern Med.

[66] Schulz KF, Altman DG, Moher D, CONSORT Group (2010). CONSORT 2010 statement: updated guidelines for reporting parallel group randomised trials. Br Med J.

[67] Alshehri MM (2018). Clinical Trials.

[68] Shafazand S, Wallace DM, Vargas SS, Del Toro Y, Dib S, Abreu AR, Ramos A, Nolan B, Baldwin CM, Fleming L (2012). Sleep disordered breathing, insomnia symptoms, and sleep quality in a clinical cohort of US Hispanics in south Florida. J Clin Sleep Med.

[69] Netzer NC, Stoohs RA, Netzer CM, Clark K, Strohl KP (1999). Using the Berlin Questionnaire to identify patients at risk for the sleep apnea syndrome. Ann Intern Med.

[70] Pereira EJ, Driver HS, Stewart SC, Fitzpatrick MF (2013). Comparing a combination of validated questionnaires and level III portable monitor with polysomnography to diagnose and exclude sleep apnea. J Clin Sleep Med.

[71] Nagappa M, Liao P, Wong J, Auckley D, Ramachandran SK, Memtsoudis S, Mokhlesi B, Chung F (2015). Validation of the STOP-Bang questionnaire as a screening tool for obstructive sleep apnea among different populations: a systematic review and meta-analysis. PLoS One.

[72] Garcia-Borreguero D, Stillman P, Benes H, Buschmann H, Chaudhuri KR, Rodríguez VM, Högl Birgit, Kohnen R, Monti GC, Stiasny-Kolster K, Trenkwalder C, Williams A, Zucconi M (2011). Algorithms for the diagnosis and treatment of restless legs syndrome in primary care. BMC Neurol.

[73] Allen RP, Stillman P, Myers AJ (2010). Physician-diagnosed restless legs syndrome in a large sample of primary medical care patients in western Europe: Prevalence and characteristics. Sleep Med.

[74] Çoban A, Yanikkerem U (2010). Sleep quality and fatigue in pregnant women. Ege Journal of Medicine.

[75] US Department of Health and Human Services, Public Health Service, National Institutes of Health, National Institute on Alcohol Abuse and Alcoholism Welcome to the MSU/COM Kobiljak Centers.

[76] Balsa AI, Homer JF, Fleming MF, French MT (2008). Alcohol consumption and health among elders. Gerontologist.

[77] Ghajarzadeh M, Sahraian MA, Fateh R, Daneshmand A (2012). Fatigue, depression and sleep disturbances in Iranian patients with multiple sclerosis. Acta Med Iran.

[78] Kang DW, Lee CU, Lim HK (2017). Role of sleep disturbance in the trajectory of alzheimer's disease. Clin Psychopharmacol Neurosci.

[79] Menza M, Dobkin RD, Marin H, Bienfait K (2010). Sleep disturbances in Parkinson's disease. Mov Disord.

[80] Wickwire EM, Williams SG, Roth T, Capaldi VF, Jaffe M, Moline M, Motamedi GK, Morgan GW, Mysliwiec V, Germain A, Pazdan RM, Ferziger R, Balkin TJ, MacDonald ME, Macek TA, Yochelson MR, Scharf SM, Lettieri CJ (2016). Sleep, sleep disorders, and mild traumatic brain injury. What we know and what we need to know: findings from a national working group. Neurotherapeutics.

[81] Ferre A, Ribó M, Rodríguez-Luna D, Romero O, Sampol G, Molina CA, Álvarez-Sabin J (2013). Strokes and their relationship with sleep and sleep disorders. Neurologia.

[82] Steinan MK, Scott J, Lagerberg TV, Melle I, Andreassen OA, Vaaler AE, Morken G (2016). Sleep problems in bipolar disorders: more than just insomnia. Acta Psychiatr Scand.

[83] Staniszewska A, Mąka A, Religioni U, Olejniczak D (2017). Sleep disturbances among patients with epilepsy. Neuropsychiatr Dis Treat.

[84] McCrae CS, Lichstein KL (2001). Secondary insomnia: diagnostic challenges and intervention opportunities. Sleep Med Rev.

[85] Jackson ML, Bruck D (2012). Sleep abnormalities in chronic fatigue syndrome/myalgic encephalomyelitis: a review. J Clin Sleep Med.

[86] Nicassio PM, Moxham EG, Schuman CE, Gevirtz RN (2002). The contribution of pain, reported sleep quality, and depressive symptoms to fatigue in fibromyalgia. Pain.

[87] Affleck G, Urrows S, Tennen H, Higgins P, Abeles M (1996). Sequential daily relations of sleep, pain intensity, and attention to pain among women with fibromyalgia. Pain.

[88] Wolfe F, Hawley DJ, Wilson K (1996). The prevalence and meaning of fatigue in rheumatic disease. J Rheumatol.

[89] Edwards RR, Cahalan C, Calahan C, Mensing G, Smith M, Haythornthwaite JA (2011). Pain, catastrophizing, and depression in the rheumatic diseases. Nat Rev Rheumatol.

[90] Gislason T, Almqvist M (1987). Somatic diseases and sleep complaints. An epidemiological study of 3,201 Swedish men. Acta Med Scand.

[91] Kim YG, Yoon DY, Kim JI, Chae CH, Hong YS, Yang CG, Kim JM, Jung KY, Kim JY (2002). Effects of health on shift-work: general and psychological health, sleep, stress, quality of life. Korean J Occup Environ Med.

[92] Atkinson TM, Mendoza TR, Sit L, Passik S, Scher HI, Cleeland C, Basch E (2010). The Brief Pain Inventory and its 'pain at its worst in the last 24 hours' item: clinical trial endpoint considerations. Pain Med.

[93] Gore M, Brandenburg NA, Dukes E, Hoffman DL, Tai K, Stacey B (2005). Pain severity in diabetic peripheral neuropathy is associated with patient functioning, symptom levels of anxiety and depression, and sleep. J Pain Symptom Manage.

[94] Beck A, Steer RA, Ball R, Ranieri W (1996). Comparison of Beck Depression Inventories -IA and -II in psychiatric outpatients. J Pers Assess.

[95] Morawetz D (2003). Insomnia and depression: which comes first?. Sleep Res Online.

[96] Cully JA, Teten AL (2008). MIRECC / CoE Home - Veterans Affairs.

[97] Cunningham JE, Shapiro CM (2018). Cognitive Behavioural Therapy for Insomnia (CBT-I) to treat depression: A systematic review. J Psychosom Res.

[98] Löwe B, Decker O, Müller S, Brähler E, Schellberg D, Herzog W, Herzberg PY (2008). Validation and standardization of the Generalized Anxiety Disorder Screener (GAD-7) in the general population. Med Care.

[99] Villarroel M, Vahratian A, Ward BW (2015). Health care utilization among US adults with diagnosed diabetes, 2013. NCHS Data Brief.

[100] Schneider ALC, Pankow JS, Heiss G, Selvin E (2012). Validity and reliability of self-reported diabetes in the Atherosclerosis Risk in Communities Study. Am J Epidemiol.

[101] Margolis KL, Lihong Q, Brzyski R, Bonds DE, Howard BV, Kempainen S, Simin L, Robinson JG, Safford MM, Tinker LT, Phillips LS, Women Health Initiative Investigators (2008). Validity of diabetes self-reports in the Women's Health Initiative: comparison with medication inventories and fasting glucose measurements. Clin Trials.

[102] White CA (2001). Cognitive behavioral principles in managing chronic disease. West J Med.

[103] Perlis M, Jungquist C, Smith MT, Posner D (2006). Cognitive Behavioral Treatment Of Insomnia: A Session-by-Session Guide.

[104] Edinger J UNC School of Medicine.

[105] Baglioni C, Spiegelhalder K, Lombardo C, Riemann D (2010). Sleep and emotions: a focus on insomnia. Sleep Med Rev.

[106] Ong JC, Shapiro SL, Manber R (2008). Combining mindfulness meditation with cognitive-behavior therapy for insomnia: a treatment-development study. Behav Ther.

[107] Conrad A, Roth WT (2007). Muscle relaxation therapy for anxiety disorders: it works but how?. J Anxiety Disord.

[108] Morin CM, Belleville G, Bélanger L, Ivers H (2011). The Insomnia Severity Index: psychometric indicators to detect insomnia cases and evaluate treatment response. Sleep.

[109] Seow LS, Abdin E, Chang S, Chong SA, Subramaniam M (2018). Identifying the best sleep measure to screen clinical insomnia in a psychiatric population. Sleep Med.

[110] Lichstein KL, Stone KC, Donaldson J, Nau SD, Soeffing JP, Murray D, Lester KW, Aguillard RN (2006). Actigraphy validation with insomnia. Sleep.

[111] Ancoli-Israel S, Martin JL, Blackwell T, Buenaver L, Liu L, Meltzer LJ, Sadeh A, Spira AP, Taylor DJ (2015). The SBSM guide to actigraphy monitoring: clinical and research applications. Behav Sleep Med.

[112] Otte JL, Payne JK, Carpenter JS (2011). Nighttime variability in wrist actigraphy. J Nurs Meas.

[113] Cole JC, Motivala SJ, Buysse DJ, Oxman MN, Levin MJ, Irwin MR (2006). Validation of a 3-factor scoring model for the Pittsburgh sleep quality index in older adults. Sleep.

[114] Fitzgerald JT, Anderson RM, Gruppen LD, Davis WK, Aman LC, Jacober SJ, Grunberger G (1998). The reliability of the Diabetes Care Profile for African Americans. Eval Health Prof.

[115] Fitzgerald JT, Davis WK, Connell CM, Hess GE, Funnell MM, Hiss RG (1996). Development and validation of the Diabetes Care Profile. Eval Health Prof.

[116] Sikaris K (2009). The correlation of hemoglobin A1c to blood glucose. J Diabetes Sci Technol.

[117] (1998). Intensive blood-glucose control with sulphonylureas or insulin compared with conventional treatment and risk of complications in patients with type 2 diabetes (UKPDS 33). UK Prospective Diabetes Study (UKPDS) Group. Lancet.

[118] Bernstein R, Parkes JL, Goldy A, Brown D, Harrison B, Chu A, Pflug BK, Simmons DA, Pardo S, Bailey TS (2013). A new test strip technology platform for self-monitoring of blood glucose. J Diabetes Sci Technol.

[119] Singh R, Kluding PM (2013). Fatigue and related factors in people with type 2 diabetes. Diabetes Educ.

[120] Zelman DC, Gore M, Dukes E, Tai K, Brandenburg N (2005). Validation of a modified version of the Brief Pain Inventory for painful diabetic peripheral neuropathy. J Vasc Nurs.

[121] Strunk Kk, Lane Fc (2016). The Beck Depression Inventory, Second Edition (BDI-II). Measurement and Evaluation in Counseling.

[122] Beck AT, Steer RA, Carbin MG (1988). Psychometric properties of the Beck Depression Inventory: Twenty-five years of evaluation. Clin Psychol Rev.

[123] Kroenke K, Spitzer RL, Williams JB, Löwe B (2010). The patient health questionnaire somatic, anxiety, and depressive symptom scales: a systematic review. Gen Hosp Psychiatry.

[124] Manber R, Edinger JD, Gress JL, Pedro-Salcedo MG, Kuo TF, Kalista T (2008). Cognitive behavioral therapy for insomnia enhances depression outcome in patients with comorbid major depressive disorder and insomnia. Sleep.

[125] Kim J, Shin W (2014). How to do random allocation (randomization). Clin Orthop Surg.

[126] Krefting D, Jansen C, Penzel T, Han F, Kantelhardt JW (2017). Age and gender dependency of physiological networks in sleep. Physiol Meas.

[127] Unruh ML, Redline S, An M, Buysse DJ, Nieto FJ, Yeh J, Newman AB (2008). Subjective and objective sleep quality and aging in the sleep heart health study. J Am Geriatr Soc.

[128] van Cauter E, Leproult R, Plat L (2000). Age-related changes in slow wave sleep and REM sleep and relationship with growth hormone and cortisol levels in healthy men. J Am Med Assoc.

